# A rare case of unilateral agenesis of left pulmonary artery presenting as severe pulmonary arterial hypertension

**DOI:** 10.21542/gcsp.2023.15

**Published:** 2023-08-01

**Authors:** Susheel Kumar Malani, Prashant Kashyap, Digvijay Nalawade

**Affiliations:** Dr. DY Patil Medical College, Pune, Maharashtra, India

## Abstract

A 26-year-old young male patient presented with progressive dyspnea over the previous 2 years. The patient also had pulmonary hypertension. Computed tomography (CT) pulmonary angiography showed absence of the left pulmonary artery, and conventional pulmonary and aortic root angiograms showed ipsilateral lung receiving collaterals from the left internal mammary artery and thyrocervical trunk.

## Introduction

Unilateral agenesis of the pulmonary artery (UAPA) is a ‘rare congenital anomaly’ that results from malformation of the sixth aortic arch on the affected side during embryogenesis^1^. The incidence of UAPA is equal in both sexes, with an annual incidence of 1 in 200,000 and an overall mortality rate of 7%^2^. It may manifest as either a single disorder or may be associated with other congenital cardiovascular malformations^3^. Approximately 30% of patients with UAPA have no cardiovascular problems; this is referred to as isolated UAPA^4^. Approximately two-thirds of isolated UAPA cases involve the right lung. Due to embryologic relationships, UAPA commonly occurs on the side of the chest, opposite the aortic arch^5^. Distal intrapulmonary branches of the affected artery usually remain intact and can be supplied by collateral vessels from the bronchial, intercostal, internal mammary, subdiaphragmatic, subclavian, or even coronary arteries^6^.

Clinical manifestations vary, and patients may not exhibit any symptoms throughout their lifespans. The manifestations that are usually observed are recurrent lung infections, reduced exercise intolerance, and shortness of breath on exertion^7^. For hemodynamic assessment, echocardiography, computed tomography (CT), and/or cardiac catheterization are required during the examination and surgical planning. The ideal management strategy for UAPA-associated congenital heart diseases includes restoring blood supply from the right ventricle to the major pulmonary artery, supplying both lungs, and repairing intracardiac shunts^8^. Here, we present the case of a 26-year-old young male with progressively worsening dyspnea who was later diagnosed with unilateral agenesis of the left pulmonary artery.

## Case summary

**Figure 1.**
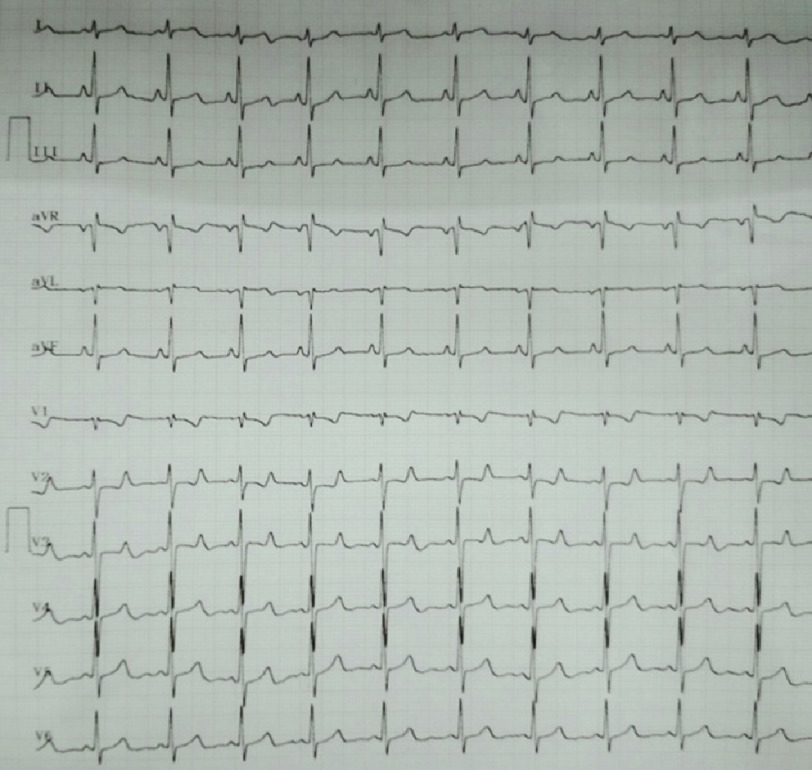
ECG revealed right axis deviation (RAD) and right bundle branch block (RBBB).

A 26-year-old young male with a body mass index of 23.45 kg/m^2^ was admitted for evaluation of progressively worsening dyspnea over the last 2 years. His baseline vitals were stable while preliminary investigations into complete blood count, renal function and liver function were normal. ECG showed right axis deviation (RAD) and right bundle branch block (RBBB) (Figure 1).

**Figure 2.**
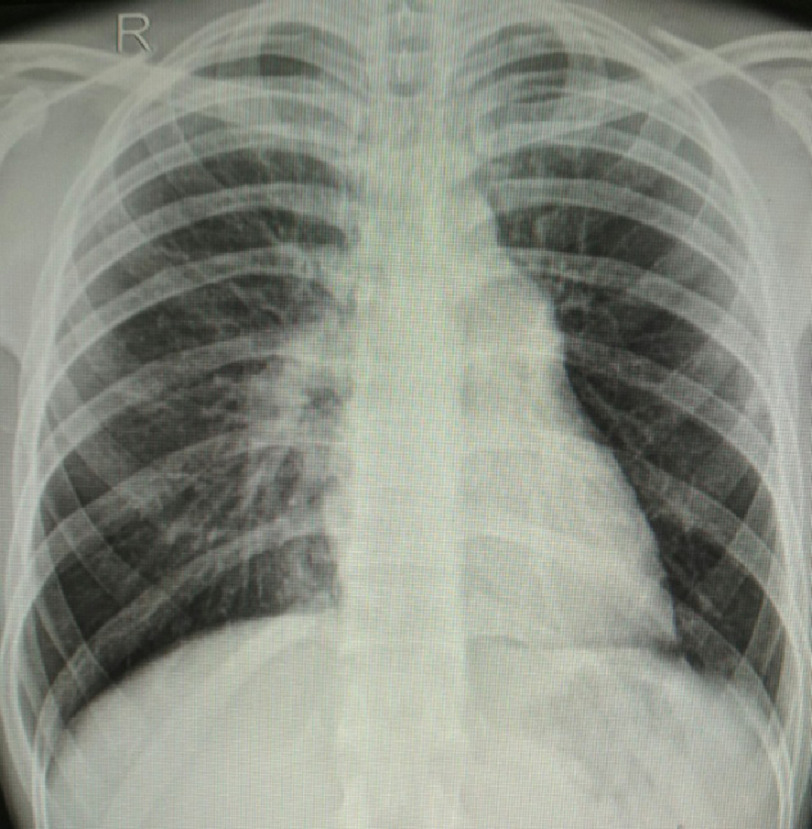
Chest radiograph showing a dilated main pulmonary artery and right pulmonary artery with reduced vascular markings in the left hemithorax.

**Figure 3.**
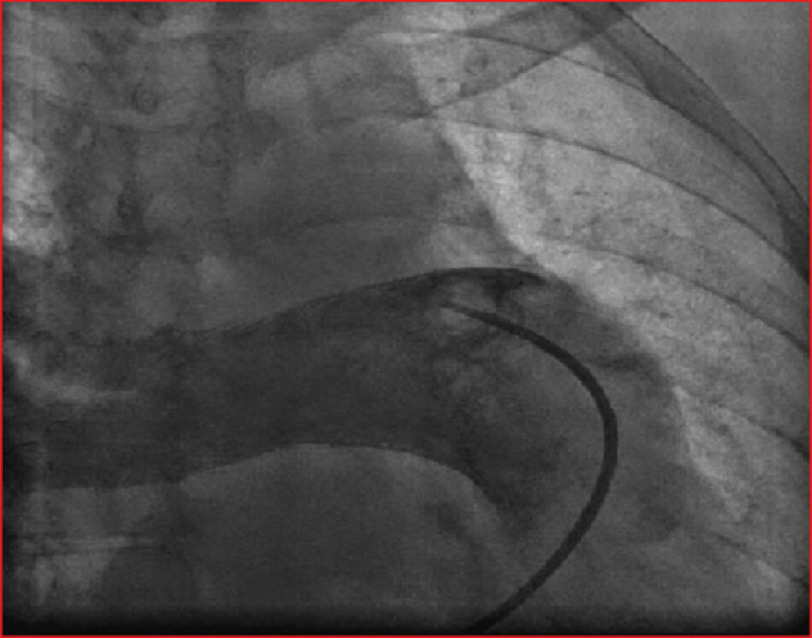
Angiography of the main pulmonary trunk with filling of the right main pulmonary artery and complete unilateral absence of left pulmonary artery (UAPA).

Creatine Kinase-Myoglobin Binding (CK-MB), D-dimer and thyroid profile were in normal range. Chest radiography showed reduced vascular marking in the left hemithorax, with a prominent right pulmonary artery and main pulmonary artery (Figure 2). Echocardiography revealed a dilated right atrium (RA) and right ventricle (RV). Moderate tricuspid regurgitation with a systolic pulmonary artery pressure (PAP) of 98 mmHg. LV function was normal, with normal mitral and aortic valves. A short-axis view at the level of the aortic valve revealed an absent left pulmonary artery. Pulmonary hypertension due to PVH and left-sided heart causes were ruled out. Transesophageal echocardiography revealed an intact interatrial septum (IAS) and an intact ventricular septum (IVS) with no intracardiac shunt. CT pulmonary angiography showed congenital interruption of the left branch of the pulmonary artery with a small left hemithorax and dilated main pulmonary artery (32 mm in diameter) and right pulmonary artery (23 mm in diameter) with no intraluminal thrombus. Subsequently, pulmonary angiography and catheterization were performed, which revealed absence of the left pulmonary artery (Figure 3).

**Figure 4.**
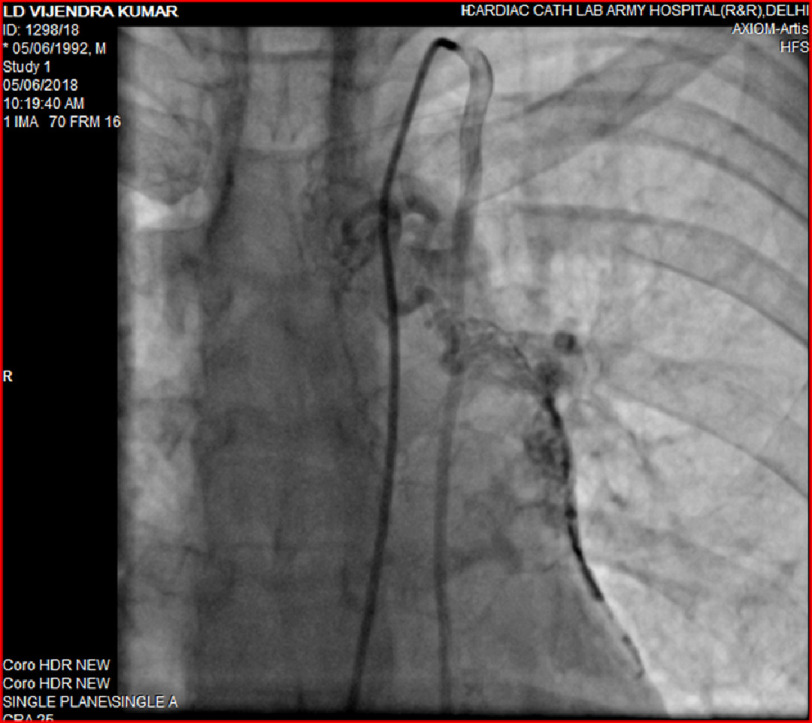
Angiogram showing prominent collaterals arising from LIMA and supplying the left pulmonary artery branches.

Pulmonary arterial pressure was 92/30 mmHg, with a mean of 56 mmHg. An aortic angiogram showed collaterals arising from the left internal mammary artery (LIMA) and thyrocervical trunk artery supplying the left-sided lung (Figure 4). Therefore, the diagnosis of an absent left pulmonary artery with distal vasculature supplied by collaterals from the left internal mammary artery and the thyrocervical trunk was confirmed. The patient was started on phosphodiesterase inhibitors (sildenafil), an endothelial receptor antagonist (bosentan), and oral anticoagulant therapy.

## Discussion

Congenital absence of one pulmonary artery is a rare birth defect which is usually observed in conjunction with cardiovascular anomalies. The right pulmonary artery (RPA) is frequently involved in agenesis^7^. In 2002, a review of 108 cases of UAPA revealed a median age at presentation of 14 years^3^. However, in our case, the age at presentation was 26 years, and agenesis of the left pulmonary artery was observed. This ailment can be underdiagnosed because 30% of the individuals do not exhibit any symptoms. This may be due to the formation of systemic collaterals in the affected lung. The prognosis depends on other associated cardiovascular anomalies and the severity of pulmonary hypertension.

Clinically, the manifestations of pulmonary artery agenesis vary. There are 2 distinct manifestations of UAPA. The first type is seen in infants and frequently manifests as congestive heart failure and pulmonary hypertension. The second type is usually observed in elderly patients and usually does not exhibit any symptoms. They tend to present with chest pain, pleural effusion, recurrent infections (37%), dyspnea or exercise intolerance (40%), pulmonary hypertension (44%), hemoptysis (20%), and high-altitude pulmonary edema (10%)^9^.

Our patient presented with progressively worsening dyspnea. Dyspnea might be due to transient elevation of pulmonary artery pressure, reduction of total lung capacity, increase in resting and/or exercise physiological dead space, and cardiac shunting^10^. Pulmonary hypertension was also observed in our patient because of an imbalance between the reduced pulmonary vascular bed caused by the absent artery and the increased blood flow to the normal pulmonary artery as the blood is directed away from the absent pulmonary artery, which causes endothelial stress and the production of vasoconstrictor mediators^7^.

Different diagnostic modalities are essential for a definitive diagnosis, but important clues are present on chest radiographs. In our case, chest radiography revealed reduced vascular marking in the left hemithorax. When suspicious findings are noted on a chest radiograph, the diagnosis of UAPA can be definitively made by CT, magnetic resonance imaging (MRI), or transthoracic echocardiogram. Echocardiography helps rule out pulmonary hypertension and other intracardiac abnormalities. Our patient had prominent collaterals originating from the left internal mammary artery and thyrocervical trunk, with no sign of myocardial ischemia.

The treatment of UAPA involves surgical, pharmacological, and behavioral management. Routine echocardiographic monitoring is recommended to detect the onset of pulmonary hypertension (PH) in asymptomatic adults. Pneumonectomy and surgical revascularization are considered in cases of recurrent hemoptysis, pulmonary infections and PH. Selective embolization of bronchial or non-bronchial systemic arteries is a valid alternative for patients with massive hemoptysis who are not eligible for surgery^1^.

Long-term vasodilator therapy, such as calcium channel blockers, prostacyclin infusion, endothelin receptor antagonists, and phosphodiesterase inhibitors, can be reserved for individuals in whom revascularization cannot be done^11^. Our patient was treated with oral anticoagulants, an endothelial receptor antagonist (bosentan), and a phosphodiesterase inhibitor(sildenafil).

### Follow-up

The patient was evaluated at one-month follow-up and there was a significant improvement from NYHA class IV to class II.

## Conclusion

Isolated unilateral pulmonary artery agenesis develops in childhood; however, it may not be identified until adulthood. Awareness of this disorder will aid in early identification and management. Collaterals to the afflicted lung from the left internal mammary artery and thyrocervical trunk are also quite unusual.

## Statement of ethics

All procedures performed in studies involving human participants were in accordance with the ethical standards of the institutional and/or national research committee and with the 1964 Helsinki declaration and its later amendments or comparable ethical standards.

## Consent

A voluntarily written informed consent was obtained from the patient to publish his case in the journal. His identity, contact details and address will not be disclosed.

## Funding declaration

The author(s) received no specific funding for this work.

## Conflicts of interest

The authors declare no conflict of interest.
